# Enhancing phytochemical parameters in broccoli through vacuum impregnation and their prediction with comparative ANN and RSM models

**DOI:** 10.1038/s41598-023-41930-8

**Published:** 2023-09-20

**Authors:** Aseeya Wahid, Saroj Kumar Giri, Adinath Kate, Manoj Kumar Tripathi, Manoj Kumar

**Affiliations:** ICAR-Central Institute of Agricultural Engineering, Bhopal, 462038 India

**Keywords:** Biochemistry, Biological techniques, Engineering, Mathematics and computing

## Abstract

Amidst increasing demand for nutritious foods, the quest for effective methods to enhance health-promoting attributes has intensified. Vacuum impregnation (VI) is a promising technique to augment produce properties while minimizing impacts on biochemical attributes. In light of broccoli’s growing popularity driven by its nutritional benefits, this study explores the impact of VI using ascorbic acid and calcium chloride as impregnation agents on enhancing its phytochemical properties. Response surface methodology (RSM) was used for optimization of the vacuum impregnation process with Vacuum pressure (0.6, 0.4, 0.2 bar), vacuum time (3, 7, 11 min), restoration time (5, 10, 15 min), and concentrations (0.5, 1.0, 1.5%) as independent parameters. The influence of these process parameters on six targeted responses viz. total phenolic content (TPC), total flavonoid content (TFC), ascorbic acid content (AAC), total chlorophyll content (TCC), free radical scavenging activity (FRSA), and carotenoid content (CC) were analysed. Levenberg–Marquardt back propagated neural network (LMB-ANN) was used to model the impregnation process. Multiple response optimization of the vacuum impregnation process indicated an optimum condition of 0.2 bar vacuum pressure, 11 min of vacuum time, 12 min of restoration time, and 1.5% concentration of solution for vacuum impregnation of broccoli. The values of TPC, TFC, AAC, TCC, FRSA, and CC obtained at optimized conditions were 291.20 mg GAE/100 g, 11.29 mg QE/100 g, 350.81 mg/100 g, 1.21 mg/100 g, 79.77 mg, and 8.51 mg, respectively. The prediction models obtained through ANN was found suitable for predicting the responses with less standard errors and higher R^2^ value as compared to RSM models. Instrumental characterization (FTIR, XRD and SEM analysis) of untreated and treated samples were done to see the effect of impregnation on microstructural and morphological changes in broccoli. The results showed enhancement in the TPC, TFC, AAC, TCC, FRSA, and CC values of broccoli florets with impregnation. The FTIR and XRD analysis also supported the results.

## Introduction

Broccoli (*Brassica oleracea* var. Italica) is a popular vegetable, as it has low calories and is rich in nutrients and antioxidants, promoting many areas of human health. Due to its excellent nutraceutical quality, broccoli alongwith cauliflower now ranks among the five eminent vegetables grown worldwide^1^.  Studies have shown that broccoli contains many bioactive chemicals and vital minerals^2^. Recent clinical trials have revealed that avoidance of severe COVID-19 symptoms can be achieved by consuming broccoli^3^. However, several biochemical processes occur in broccoli during post-harvest leading to shorter life.

The opening of florets, loss of stem firmness and green colour, surface drying, formation of foul odours, and soft rots are just a few reactions that, if unchecked, can cause unacceptable nutrient and quality loss^4^. Controlled refrigerated environment storage, thermal shock treatment, modified atmosphere packaging, application of plant hormones, chemical treatments, light irradiation, and other methods have all been used to extend the postharvest life and preserve the quality of broccoli. However, a less expensive, risk-free, and nontoxic approach is required to be developed that can increase broccoli’s quality and shelf life.

Vacuum impregnation (VI) is one approach for enhancing food products’ quality with beneficial nutrients and changing their sensory and physicochemical attributes. VI is considered an effective technique for introducing required solutes and solvents into the porous microstructure of the food matrix due to a mechanically generated pressure gradient. Numerous chemical processes are catalysed by hydrodynamic mechanisms and deformation-relaxation events induced by pressure fluctuations, which help this process^5,6^. Despite its advantages, VI technology does have drawbacks, including issues with regulating mass transfer rates, not able of reusing solutions, high cost and selecting suitable reagents for impregnation. These obstacles highlight the necessity for extensive research to understand and overcome them^7^.

In the vacuum impregnation process, a vacuum is generated over raw food items submerged in a solution of particular chemical compounds, which removes any native liquids or gases that previously filled the porosity area. Thereafter, the relaxation phase occurs when atmospheric pressure is recovered; subsequently, the solution is injected into the capillaries and pores of biological tissue. Under lowered pressure, the solutes replace the air within the pores, reducing oxidation reaction and prolonging the product’s shelf life. Traditional impregnation at atmospheric pressure might take days, whereas VI can be performed in less time and significantly reduce ravage of the food’s nutrients and structure. It has been utilized extensively in the food sector to reduce the soaking time of foods, boost their nutritional content, and accelerate the osmotic dehydration process^8^. To this extent we know, there is insufficient literature concerning the impacts of VI with different agents on the biochemical qualities of broccoli, and optimization of the VI process. We investigated the possible application of vacuum impregnation with ascorbic acid and calcium chloride (CaCl_2_) in improving the nutritional quality of broccoli. The aim of the study was to find a suitable condition for vacuum impregnation of broccoli florets through optimization of process parameters by using RSM and ANN techniques.

## Materials and methods

### Chemicals and materials

Different chemical reagents used for analysis viz. ascorbic acid, calcium chloride, quercetin, aluminium chloride, sodium nitrite, sodium hydroxide pellets, gallic acid, folin-ciocalteu phenol reagent, anhydrous sodium carbonate, 2,2-diphenyl-1-picrylhydrazyl (DPPH), sodium bicarbonate, methanol, tri-chloro acetic acid, and acetone were of analytical grade and purchased from Sigma-Aldrich (St. Louis, USA) and Himedia (HiMedia Laboratories, Mumbai, India). Broccolis (cultivar: Pusa KTS-1) utilized in the study were obtained from the Farm section of ICAR-Central Institute of Agricultural Engineering in Bhopal, India (23.3156° N, 77.4037° E). The broccolis were harvested 125 days after sowing of seeds and 90 days of transplanting. Mass of individual heads were in the range of 350–450 g.

### Sample preparation

The individual florets were cut and separated from the broccoli head with a sharp knife, and washed with running tap water. The washed florets were utilized for the experiments of vacuum impregnation. All methods regarding plant sample collections were carried out with permission from the Institute and in accordance with national guidelines.

### Vacuum impregnation process

**Figure 1:**
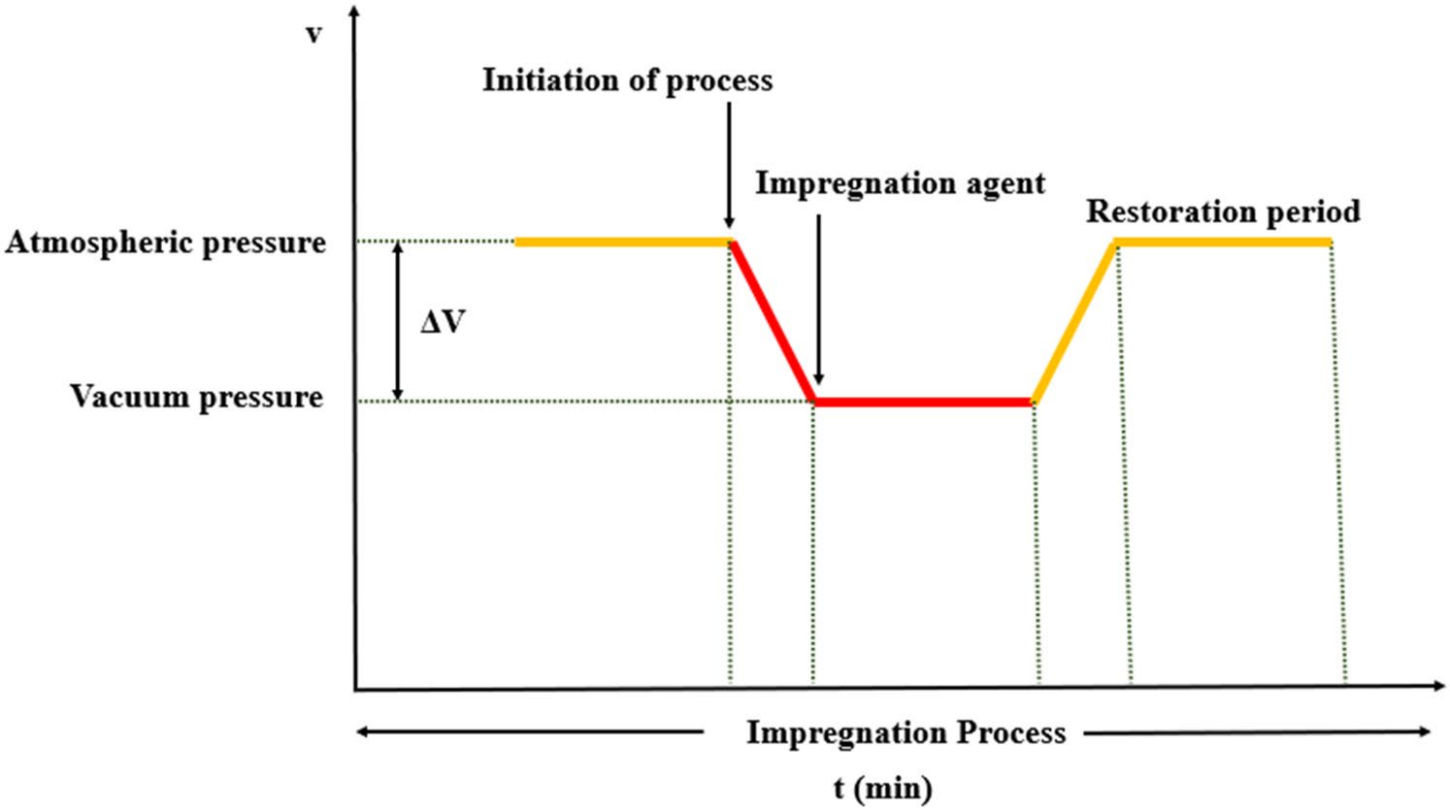
Schematic diagram of vacuum impregnation process.

The laboratory model vacuum impregnation system developed at ICAR-Central Institute of Agricultural Engineering (India) was used for the study. The system consists of a vacuum chamber, vacuum pump and an automation setup with sensors to set and monitor the desired level of vacuum inside the chamber. During experimentation, around 15 g sample of broccoli florets were placed in a glass beaker of 1000 ml capacity. The beaker was then placed in the impregnation chamber and subjected to the set values of vacuum pressures as per the experimental conditions. The sample was then submerged in and simultaneously kept under vacuum for a specified time (vacuum time). Once the vacuum period was achieved, atmospheric pressure was restored and sample was kept under atmospheric condition for a specified period (restoration time). The whole process is shown in Fig. 1. Finally, the impregnation solution over the surface of florets was extracted, lightly dried on filter paper, carefully weighed (to within 0.001 g), and evaluated for quality. Different concentrations of impregnation solutions containing a mixture of calcium chloride (CaCl_2_) and ascorbic acid were utilized for the study.

### Experimental procedure

The range of process variables viz. vacuum pressure, vacuum time, restoration time, and concentration was selected based on some preliminary experiments. Response surface methodology’s (RSM) Box Behnken design (BBD) was utilized to comprehend the outcome of four input variables, namely vacuum pressure (VP), vacuum time (Vt), restoration time (Rt) and Concentration (Cn) on the responses, including total phenolic content (TPC), total flavonoid content (TFC), ascorbic acid content (AAC), total chlorophyll content (TCC), free radical scavenging activity (FRSA), and carotenoid content (CC). Experiments were conducted using the BBD design with 29 experiments and 5 centre points (Table 1). Three replications were taken to determine the pure error.

**Table 1:** Experimental design and quality parameters of vacuum impregnated broccoli florets.

S. no.	Vp (bar)	Vt (Min)	Rt (Min)	Cn (%)	TPC (GAE/100 g FW)	TFC (mg/100 g FW)	AAC (mg/100 g FW)	TCC (mg/g FW)	FRAS (%)	CC (mg/g FW)
1	0.60	11.00	10.00	1.00	225.14 ± 0.43	10.01 ± 0.46	251.50 ± 0.64	0.83 ± 0.47	70.79 ± 0.56	7.08 ± 0.36
2	0.60	7.00	5.00	1.00	194.97 ± 0.53	9.92 ± 0.12	164.32 ± 0.27	0.80 ± 0.53	64.77 ± 0.77	7.58 ± 0.34
3	0.60	3.00	10.00	1.00	191.15 ± 0.67	10.09 ± 0.78	168.19 ± 0.21	0.72 ± 0.79	78.79 ± 0.18	8.40 ± 0.34
4	0.60	7.00	15.00	1.00	256.32 ± 0.35	9.63 ± 0.12	207.46 ± 0.17	0.65 ± 0.56	74.77 ± 0.30	6.80 ± 0.22
5	0.20	7.00	5.00	1.00	240.91 ± 0.47	9.43 ± 0.25	259.29 ± 0.47	0.95 ± 0.43	78.79 ± 0.12	8.64 ± 0.12
6	0.20	3.00	10.00	1.00	226.54 ± 0.92	9.51 ± 0.34	232.33 ± 0.22	0.99 ± 0.71	64.77 ± 0.19	9.80 ± 0.30
7	0.20	11.00	10.00	1.00	189.12 ± 0.20	10.23 ± 0.71	327.58 ± 0.21	0.93 ± 0.53	78.79 ± 0.12	8.18 ± 0.09
8	0.20	7.00	15.00	1.00	211.78 ± 0.25	9.47 ± 0.25	288.46 ± 0.28	0.90 ± 0.84	64.77 ± 0.17	8.84 ± 0.42
9	0.40	3.00	5.00	1.00	213.29 ± 0.38	9.40 ± 0.74	211.82 ± 0.53	0.82 ± 0.29	78.79 ± 0.27	8.65 ± 0.22
10	0.40	11.00	5.00	1.00	120.75 ± 0.56	9.56 ± 0.65	267.03 ± 0.07	0.92 ± 0.78	64.77 ± 0.24	7.04 ± 0.25
11	0.40	7.00	10.00	1.00	168.05 ± 0.83	9.10 ± 0.13	286.67 ± 0.40	0.87 ± 0.11	78.79 ± 0.21	6.18 ± 0.17
12	0.40	7.00	10.00	1.00	149.13 ± 0.21	9.13 ± 0.22	235.88 ± 0.31	0.84 ± 0.29	74.77 ± 0.17	6.01 ± 0.31
13	0.40	7.00	10.00	1.00	178.36 ± 0.21	9.18 ± 0.30	229.10 ± 0.22	0.88 ± 0.21	78.79 ± 0.21	6.50 ± 0.65
14	0.40	7.00	10.00	1.00	173.32 ± 0.20	9.09 ± 0.31	274.79 ± 0.31	0.93 ± 0.03	79.77 ± 0.22	6.50 ± 0.34
15	0.40	7.00	10.00	1.00	161.54 ± 0.46	9.13 ± 0.12	251.10 ± 0.04	0.91 ± 0.12	78.79 ± 0.17	6.30 ± 0.48
16	0.40	3.00	15.00	1.00	114.19 ± 0.54	9.84 ± 0.16	187.70 ± 0.23	0.75 ± 0.03	64.77 ± 0.12	7.70 ± 0.12
17	0.40	11.00	15.00	1.00	251.18 ± 0.18	9.16 ± 0.47	372.86 ± 0.23	0.88 ± 0.38	78.79 ± 0.25	5.87 ± 0.44
18	0.60	7.00	10.00	1.50	202.82 ± 0.78	9.83 ± 0.11	238.75 ± 0.33	0.72 ± 0.11	74.77 ± 0.20	6.04 ± 0.17
19	0.40	7.00	5.00	1.50	197.08 ± 0.03	9.77 ± 0.03	195.85 ± 0.21	0.91 ± 0.31	78.79 ± 0.41	4.99 ± 0.30
20	0.40	3.00	10.00	1.50	164.07 ± 0.33	9.36 ± 0.07	178.89 ± 0.12	0.95 ± 0.09	64.77 ± 0.21	6.58 ± 0.42
21	0.40	11.00	10.00	1.50	223.35 ± 0.54	9.99 ± 0.12	335.07 ± 0.17	0.99 ± 0.29	78.79 ± 0.34	5.52 ± 0.28
22	0.40	7.00	15.00	1.50	201.72 ± 0.29	9.11 ± 0.18	304.09 ± 0.02	0.93 ± 0.22	64.77 ± 0.71	5.40 ± 0.54
23	0.20	7.00	10.00	0.50	195.68 ± 0.54	9.71 ± 0.08	317.48 ± 0.16	0.76 ± 0.30	78.79 ± 0.37	6.50 ± 0.05
24	0.20	7.00	10.00	0.50	197.52 ± 0.02	10.58 ± 0.28	385.46 ± 0.22	0.71 ± 0.71	74.77 ± 0.16	7.30 ± 0.17
25	0.60	7.00	10.00	0.50	284.75 ± 0.22	11.95 ± 0.34	248.66 ± 0.12	0.92 ± 0.28	75.79 ± 0.27	6.64 ± 0.23
26	0.40	7.00	5.00	0.50	229.16 ± 0.12	9.98 ± 0.14	315.80 ± 0.33	0.87 ± 0.12	64.77 ± 0.20	6.27 ± 0.02
27	0.40	3.00	10.00	0.50	211.84 ± 0.07	12.03 ± 0.08	238.03 ± 0.26	0.84 ± 0.11	78.79 ± 0.23	8.55 ± 0.47
28	0.40	11.00	10.00	0.50	204.88 ± 0.44	10.39 ± 0.14	351.79 ± 0.12	0.88 ± 0.25	64.77 ± 0.22	3.50 ± 0.02
29	0.40	7.00	15.00	0.50	244.30 ± 0.13	10.91 ± 0.28	336.46 ± 0.46	0.80 ± 0.25	78.79 ± 0.12	4.07 ± 0.25

### Biochemical analysis

The analysis of biochemical parameters, including the quantification of total phenolic content (TPC), total flavonoids content (TFC), total ascorbic acid (AAC), free radical scavenging activity (FRSA), total chlorophyll content (TCC) and carotenoid content (CC), were carried out on triplicates of broccoli samples following a series of methods.

Initially, 0.5 g of fresh broccoli florets were combined with an 80% methanolic solution and subjected to centrifugation at 553.41 G for 20 min using a C 24 Remi Group Laboratory Instruments centrifuge (India). The resulting supernatant was obtained by filtering the methanolic extract through qualitative filter paper (100,125 R, AXIVA SICHEM BIOTECH, India)^9,10^. The methanolic extract was further used for the TPC, TFC and FRSA analysis.

The estimation of TPC was followed in which a 0.2 mL volume of the methanolic extract was mixed with 10 mL of Folin–Ciocalteu reagent (diluted tenfold), followed by adding 8 mL of 7.5% sodium carbonate. After incubating in darkness for 90 min, the absorbance at 765 nm was measured. A gallic acid equivalent (GAE) per 100 g of fresh weight was calculated based on the obtained values^11^. The TFC of florets was estimated as a volume of mL methanolic extract 510 nm using the same extraction as that of phenolic content^12^, and expressed in mg of quercetin equivalent/100 g fresh weight. The FRSA of the sample was determined using DPPH assay at 517 nm as described by Erken & Kaya^12^ and expressed as per cent. The extraction procedure for ascorbic acid estimation was adopted from Rajoriya et al. ^11^. Afterward, 10 mL of extract was mixed with 2 mL of tri-chloro acetic acid (10%) and incubated in ice bath for 5 min. Then, 2 mL of Folin–Ciocalteu (tenfold diluted) reagent was added to the mixture, and the absorbance was measured at 760 nm using a spectrophotometer (UV-1800, Shimadzu, Japan)^11^. The chlorophyll concentration (mg/g of fresh weight) was determined using a method described by Albanese et al.^13^ with some modifications, where the absorbance was measured at 645 and 663 nm. The carotenoids content (mg/g) was calculated as per the procedure described by Seely et al.^14^ at 480 nm and 510 nm.

### Instrumental characterization

Fresh (control without vacuum impregnated) and vacuum impregnated broccoli florets at the optimized conditions were freeze-dried and pulverized with an analytical mill (model: 4301-02, Cole-Parmer Instrument Co., India). The acquired dried samples were sieved (350–200 m: BSS sieve 50 and 70) and then used for analysis regarding changes in morphology, structures and chemical bonds.

### X-ray diffraction

X-ray diffraction equipment (XRD, Bruker D8 Advance, Germany) with Cuka (= 1.5406 and step size = 0.02°) radiation at the scattering edge run 5–60 was utilized to determine the structural analysis and phase composition of the products at room temperature^15^. The particle size was calculated using the Debye–Scherrer formula:

$${\mathrm{D}}\, = \,\left( {0.{9}\lambda } \right)/ \, \left( {\beta {{\cos }}\theta } \right)$$

Where ‘λ’ is the X-ray wavelength, ‘β’ is the FWHM (full width at half maximum), ‘θ’ is the diffraction angle, and ‘D’ is the diameter of the particle^16^.

### Field emission scanning electron microscopy

The secondary electron mode of the FE-SEM (Carl Zeiss, Ultra Plus) was used to observe the surface microstructure of treated and untreated broccoli. The sample was immobilized on specimen stubs and coated with gold. A small amount of freeze-dried broccoli florets was evenly dispersed on a stage and fixed with double-sided tape. Gold preprocessing was performed under vacuum conditions prior to observing under the SEM, at an accelerating voltage of 5.0 kV.

### Fourier transform infrared spectroscopy (FTIR)

The FTIR spectra was analysed using an FTIR spectrometer (NICOLET 6700, Thermo Fisher Scientific, Germany). For the IR spectrum, a small amount of powder was mixed with KBr and Pellet (pressed-disk) technique was used for this purpose. With an average of 64 scans, the infrared absorption of thin-film between 4000 and 400 cm^−1^ was measured. All spectra were taken at room temperature and under natural light.

### ANN modelling

The Neural Network feature of the MATLAB R2020a was used to simulate the vacuum impregnation process for development of an ANN model to represent the process. Accordingly, the dependent variable models were constructed using the back-propagation process, and 87 data sets were utilized to guarantee that the ANN was adequately modelled. Trial and error method was used for obtaining optimal number of neurons within the hidden layer. The model was executed to prevent overfitting. Consequently, the 4-10-1 architecture of ANN was used for the study, where four input variables viz. VP, Vt, Rt, and Cn, ten neurons in the hidden layer, and one output variable among the selected dependent variables, one at a time. The back-propagation model utilized the Levenberg-Marquadt algorithm; hence, the training function with gradient descent was used to minimize the sum of square errors (MSE) of the network^17,18^. Training dataset accounted for 70%, whereas 30% was used for validation and testing. The training dataset investigated the link between the network’s input and output properties. The validation dataset was crucial for network generalization, whereas the testing dataset contributed to network predictability. Typically, assigning more data to the training reduces processing time while enhancing the model accuracy^19^.

### RSM modelling and optimization

The second-order polynomial model was developed for each dependent variable to interpret the individual and interactive effect of the independent variables^10^. The adequacy of the developed model was checked through the estimation of model parameters like F test (*p* ≤ 0.05), coefficient of determination (R^2^), lack-of-fit, and coefficient of variation (c.v. 10%). The visual representation of the developed second-order model was carried out through 3D surface plot. Analysis of variance (ANOVA) were used to describe the correlations between the responses and independent factors with their significance of effect. Further, the pre-set goal based (both for independent and dependent variables) optimization of independent variables were done using Design Expert (version 13, student version) software. All of the responses and independent variables were treated equally (+ + +) during setting of the goal and optimization. The desirability function Dr(x) was computed, and the point with the most significant desirability was chosen as the optimal numerical solution.

### Model comparisons

The RSM and ANN models were compared based on statistical analysis. There were six standard error functions used likewise Chi-square (χ^2^), Residual Sum of Squares Error (ERRSQ/SSE), Coefficient of determination (R^2^), Hybrid Fractional Error Function (HYBRID), Average Relative Error (ARE), Root Mean Square Error (RMSE, polynomial) and Marquardt’s Percent Standard Deviation (MPSD). The equations for error analysis are as follows:

$${\mathrm{SSE}} = \mathop \sum \limits_{i = 0}^{n} \left({{D_{p}} - {D_{e}}}\right)^{2}$$

$${\mathrm{Chi}} - {\mathrm{square}} = \mathop \sum \limits_{i = 0}^{n} \frac{{\left( {{D_{p}} - {D_{e}} } \right)^{2} }}{{{D_{e}}}}$$

$${\mathrm{HYBRID}} = \frac{100}{{N - p}} .\mathop \sum \limits_{i = 1}^{n} \frac{{{D_{e}} - {D_{p}} }}{{D_{e} }}$$

$${\mathrm{MPSED}}\;\left( \% \right) \, = \sqrt {\frac{{\sum \left( {\frac{De - Dp}{{D{\mathrm{e}}}}} \right)^{2} }}{N - P}} \times 100$$

$${\mathrm{RMSE}} = \sqrt {\frac{{\mathop \sum \nolimits_{n - 1}^{n} \left( {{D_{e}} - {D_{p}} } \right)^{2} }}{N}}$$

$${\mathrm{ARE}}\; \, \left( \% \right) \, = \frac{100}{N}\mathop \sum \limits_{i = 1}^{n} \frac{{{D_{e}}- {D_{p}} }}{{D_{e} }}$$

## Results and discussion

### Effect of vacuum impregnation process variables on quality parameters

**Figure 2:**
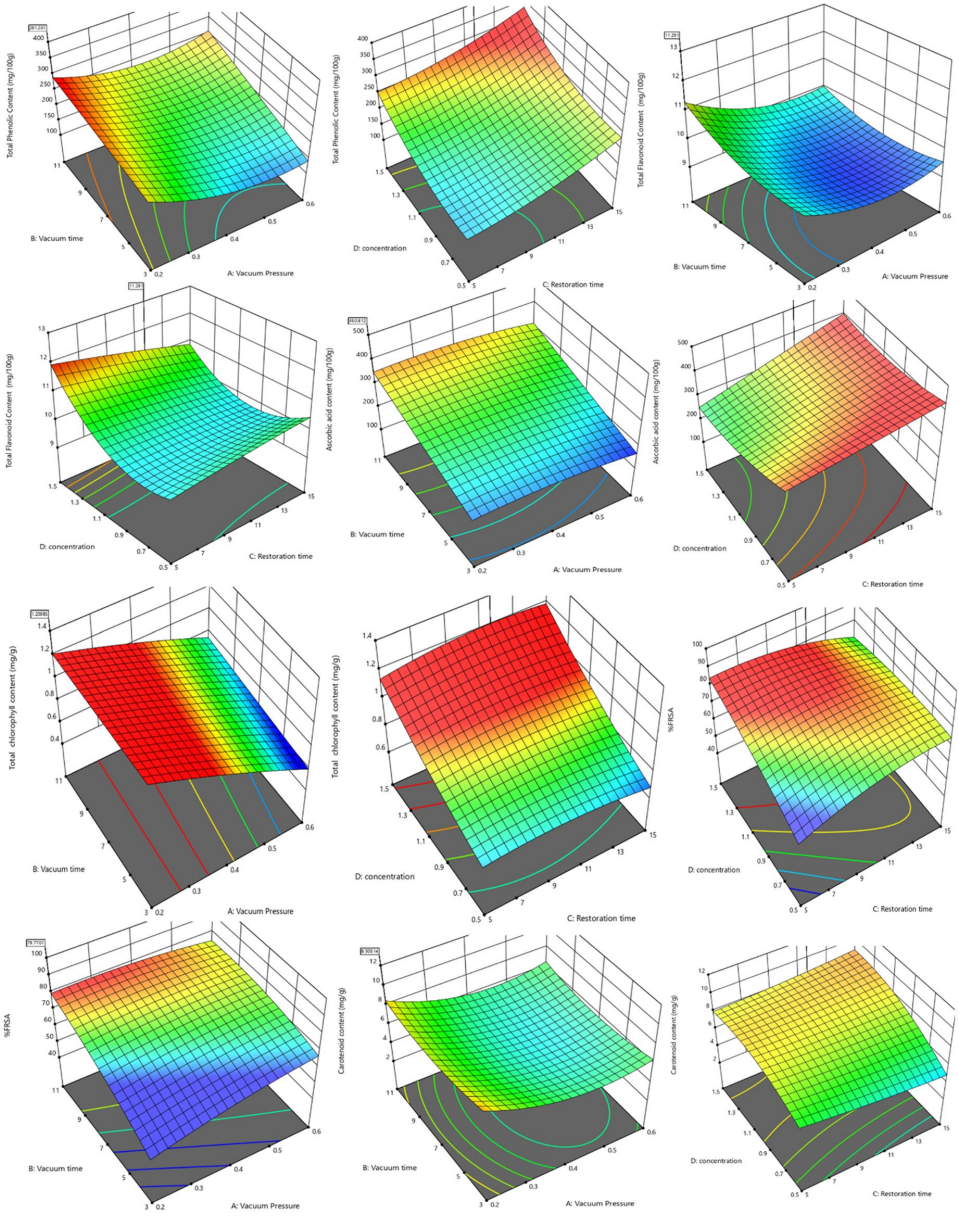
Effect of independent parameters on the biochemical properties of broccoli florets: Graphs (**A** & **B**) represent effect on TPC; (**C** & **D**) represent effect on TFC; (**E** & **F**) represent effect on AAC; (**G** & **H**) represent effect on TCC; (**I** & **J**) represent effect on FRSA; (**K** & **L**) represent effect on CC.

The values of different responses for broccoli samples obtained at various experimental conditions are presented in Table 1. The second-order polynomial model was fitted to each response data independently. The model represents the effect of various independent variables and their possible interactions on the response value. The graph of residuals’ normal probability dropped in a straight line, indicating that the errors are normally distributed. The normal plot of residuals and the external residual plot were used to compare the diagnostic relationship between the experimental data and the predicted data. The residuals points were tightly spaced along the straight line in the normal plot, with just a few places respectfully positioned outside the diagonal, demonstrating that the residuals were normally distributed. The 3D plots of the second order model of each dependent variables are shown in Fig. 2. The model equations in the form of actual factor for predicting all responses are given below:

$$\begin{aligned} {\mathrm{TPC}} = & {743.67} - {42.26}\;{\mathrm{VT}} - 169.45 \;{\mathrm{Cn}} + {22.32}\;{\mathrm{VP}}*{\mathrm{VT}} + {22.62}\;{\mathrm{ VP}}*{\mathrm{RT}} - {420.72}\;{\mathrm{VP}}*{\mathrm{Cn}} \\ & + {2.87}\;{\mathrm{VT}}*{\mathrm{RT}} + 8.28\;{\mathrm{VT}}*{\mathrm{Cn}} + {1041.39}\;{\mathrm{VP}}^{2} + 0.623\;{\mathrm{RT}}^{2} + 136.85\;{\mathrm{Cn}}^{2} \\ \end{aligned}$$

$$\begin{aligned} {\mathrm{TFC}} = & 12.77 - \, 0.215\;{\mathrm{VP}} - {6.28}\;{\mathrm{Cn}} - {6.19}\;{\mathrm{VP}}*{\mathrm{Cn}} + 0.28\;{\mathrm{VT}}*{\mathrm{Cn}} \\ & - \, 0.159\;{\mathrm{RT}}*{\mathrm{Cn}} + {12.43}\;{\mathrm{VP}}^{{2}} + 0.024\;{\mathrm{VT}}^{{2}} + {3.61}\;{\mathrm{Cn}}^{{2}} \\ \end{aligned}$$

$$\begin{aligned} {\mathrm{AAC}} = & {611.87} - {84.25}\;{\mathrm{VP}} - {6}.{21}\;{\mathrm{VT}} - {16}.{11}\;{\mathrm{RT}} - {494.37}\;{\mathrm{Cn}} \\ & + {1.624}\;{\mathrm{VT}}*{\mathrm{RT}} + {8.76}\;{\mathrm{RT}}*{\mathrm{Cn}} + 115.80\;{\mathrm{Cn}}^{{2}} \\ \end{aligned}$$

$$\begin{aligned} {\mathrm{TCC}} = & \, 0.2848 + {1.59}\;{\mathrm{VP }} - 0.0{161}\;{\mathrm{VT}} + 0.026\;{\mathrm{RT}} + 0.481\;{\mathrm{Cn}} + 0.053\;{\mathrm{VP}}*{\mathrm{ VT}} \\ & - 0.0257\;{\mathrm{VP}}*{\mathrm{RT}} + 0.000327\;{\mathrm{VT}}*{\mathrm{RT}} - \, 0.00164\;{\mathrm{RT}}^{{2}} + 0.122\;{\mathrm{Cn}}^{{2}} \\ \end{aligned}$$

$$\begin{aligned} {\mathrm{FRSA }}\left( \% \right) \, = & 66.049 - 6.88125\;{\mathrm{VP}}*{\mathrm{VT}} + 6.005\;{\mathrm{VP}}*{\mathrm{RT}} + 0.3505\;{\mathrm{VT}}*{\mathrm{RT}} + 3.505\;{\mathrm{VT}}*{\mathrm{Cn}} \\ & - 2.804\;{\mathrm{RT}}*{\mathrm{Cn}} - 0.193\;{\mathrm{VT}}^{{2}} - 0.174\;{\mathrm{RT}}^{{2}} - 8.634\;{\mathrm{Cn}}^{{2}} \\ \end{aligned}$$

$$\begin{aligned} {\mathrm{CC}} = & {18.295} - 27.278\;{\mathrm{VP}} - {1.438}\;{\mathrm{VT}} - \, 0.327\;{\mathrm{RT}} + \, 0.498\;{\mathrm{VT}}*{\mathrm{Cn}} \\ & + \, 0.2605\;{\mathrm{RT}}*{\mathrm{Cn}} + {36.915}\;{\mathrm{VP}}^{{2}} + 0.0477\;{\mathrm{VT}}^{{2}} - 4.305\;{\mathrm{Cn}}^{{2}} \\ \end{aligned}$$

### Total phenolic content

The resilience that phenolic compounds confer to horticultural products to a variety of postharvest quality-degrading situations is a significant factor in the shelf life of the produce^20^. Fresh broccoli florets contained 115.48 mg GAE/100 g FW TPC. TPC of broccoli florets increased due to impregnation, with the maximum rise up to 284.75 mg GAE/100 g FW for the sample treated at 0.6 bar VP, 7 min VT, 10 min RT, and 0.5% Cn. VT and Cn had a significant effect on the phenolic content of broccoli florets, as indicated by the model’s coefficient of determination (R^2^) of 0.95 (Table 2). TPC showed an increasing trend with VT, RT, Cn, whereas it declined in content with an increase in vacuum pressure (Fig. 2). Broccoli florets exhibited an increase in TPC may be due to moisture removal, which led to phenols accumulating and becoming more detectable via phenolic content analysis. In addition, applying a vacuum on the florets may have resulted in the rupture of the cell wall, releasing intracellular phenolic acids and increasing TPC. Also, phenolic compounds and oxygen may not have been able to come in contact due to the hydrodynamic mechanism of vacuum infusion. In contrast, the inactivation of PPO could have reduced the oxidative reactions of phenols. As a result of vacuum penetration, cellular constituents may have been broken down, increasing the accessibility of active components during extraction. Finally, the defence mechanism of phenolic contents against various stresses, such as vacuum, could have also contributed to the accumulation of TPC^21–23^.

**Table 2:** Analysis of variance (ANOVA) for response variables.

	TPC	TFC	AsA	TCC	FRSA	CC
Model F-value	19.47	12.94	11.38	12.01	17.2	19.35
Standard deviation	12	0.2868	24.58	0.0347**	1.94	0.4503
Mean	200.79	9.84	264.22	0.857	73.86	6.81
Cv. %	5.98	2.91	9.3	4.04	2.63	6.61
*p-values*						
Model	0.0001*	0.0001*	0.0001*	0.0001*	0.0001*	0.0001*
A-vacuum pressure	0.571	0.0418**	0.0003*	0.0001*	0.8922	0.0005*
B-vacuum time	0.0414**	0.3954	0.0001*	0.0067*	0.5965	0.0001*
C-restoration time	0.0648	0.9485	0.005*	0.0119**	0.6671	0.0123**
D-concentration	0.0405**	0.0001*	0.0026*	0.0001*	0.4801	0.8383
A*B	0.01**	0.1903	0.8116	0.0279**	0.0001*	0.7368
A*C	0.0021*	0.5822	0.7804	0.1599	0.0003*	0.2943
A*D	0.0001*	0.0025*	0.1723	0.0001*	0.4886	0.194
B*C	0.0001*	0.1608	0.0193**	0.7113	0.0001**	0.8112
B*D	0.0154**	0.0014*	0.4026	0.9983	0.0001**	0.0006*
C*D	0.6685	0.0148**	0.0965	0.2153	0.0001**	0.0118**
A^2^	0.0001*	0.0008*	0.1425	0.1802	0.1333	0.0001*
B^2^	0.6904	0.0045*	0.9532	0.977	0.0011*	0.0008*
C^2^	0.0056*	0.7454	0.93	0.0099*	0.0002*	0.4587
D^2^	0.0001**	0.0001**	0.0123**	0.0497**	0.0107**	0.0001**
R^2^	0.9511	0.9283	0.9192	0.9232	0.945	0.9509
Adjusted R^2^	0.9023	0.8565	0.8385	0.8463	0.8901	0.9017
Adeq. precision	20.0397	13.9885	12.3863	12.971	11.4494	19.6403
Lack of fit *p*-value	0.3113	0.4762	0.8703	0.5375	0.6955	0.1524

Significant at *p* < 0.01; ** significant at *p* < 0.05.

### Total flavonoid content

Flavonoids are secondary metabolites having antioxidant activity, the efficacy of which is dependent on the location and number of free hydroxyl (OH) groups^24^. Fresh broccoli florets contained 7.94 mg/100 g FW flavonoids. A significant increase in flavonoid content of 11.95 mg/100 g FW was obtained for the combination of 0.6 bar VP, 7 min VT, 10 min RT, and 0.5% Cn during the impregnation of broccoli. The model’s coefficient of determination (R^2^) was 0.93 (Table 2). The effects of vacuum pressure and concentration on TFC were significant. Concentration and vacuum pressure significantly affected TFC, which showed an accelerating trend with concentration and declined in content with increase in VP (Fig. 2). The increase in flavonoid content of impregnated broccoli florets may be due to the hydrodynamic mechanism effect of vacuum impregnation^24^.

### Ascorbic acid content

Ascorbic acid is a critical nutrient more sensitive to oxidative degradation during food preparation and storage than other nutrients^25^. Fresh broccoli florets contained 111.38 mg/100 g FW of ascorbic acid. A significant rise in AAC up to 385.46 mg/100 g FW was obtained due to impregnation at the experimental condition of 0.2 bar VP, 7 min VT, 10 min RT, and 0.5% Cn. As one of the impregnating agents, the ascorbic acid content was significantly affected by all independent parameters (Table 2). Ascorbic acid content showed increasing trend with vacuum time and restoration time. It declined with increasing vacuum pressure and concentration (Fig. 2).

### Total chlorophyll content

Changes in chlorophyll concentration in photosynthetic cells are an excellent diagnostic of senescence in green vegetables after harvest. An increase in membrane permeability has been connected to chlorophyll depletion^26^. Fresh broccoli florets had TCC of 0.40 mg/g FW. The increase in TCC was recorded with decreasing vacuum pressure and increasing concentration of the solution. TCC data was fitted with a polynomial model with coefficient of determination (R^2^) value of 0.92. Each independent parameter had a considerable impact on TCC (Table 2). TCC has shown a linear increasing trend with concentration of the impregnating solution, vacuum time as well as restoration time, and linearly decreasing trend with vacuum pressure. Increase in chlorophyll content may be due to the synergetic effect of calcium chloride and ascorbic acid as both have known to prevent degradation and neutralize radicals^27^.

### Free radical scavenging activity

An essential criterion for determining the nutritional content of fruits and vegetables is the total antioxidant capacity. Due to its high phenolic content, broccoli has the potential to be a nutritious food with rich in antioxidant activity. It also contains a combination of additional antioxidants identified to delay the onset of chronic diseases, such as carotenoids, ascorbic acid, and α-tocopherol^28^. Fresh broccoli had an FRS activity of up to 59.10%. Broccoli’s FRS activity increased to a maximum value of 78.79% after impregnation under different combinations of independent variables. The coefficient of determination (R^2^) of the model was obtained as 0.94 (Table 2). The independent parameters did not significantly impact on the changes in FRSA. The increment in scavenging activity may be due to increment in phenol, flavonoid and ascorbic acid content.

### Carotenoid content

Fresh broccoli florets had 6.38 mg/g FW of carotenoids. Broccoli’s carotenoid content increased maximum up to 9.84 mg/g after impregnation under the impregnation conditions 0.2 bar VP, 3 min VT, 10 min RT, and 1% Cn. The model’s R^2^ was 0.95. The parameters VP, VT, and RT significantly influenced the CC (Table 2).

### Prediction of responses with artificial neural network (ANN) modelling

**Figure 3:**
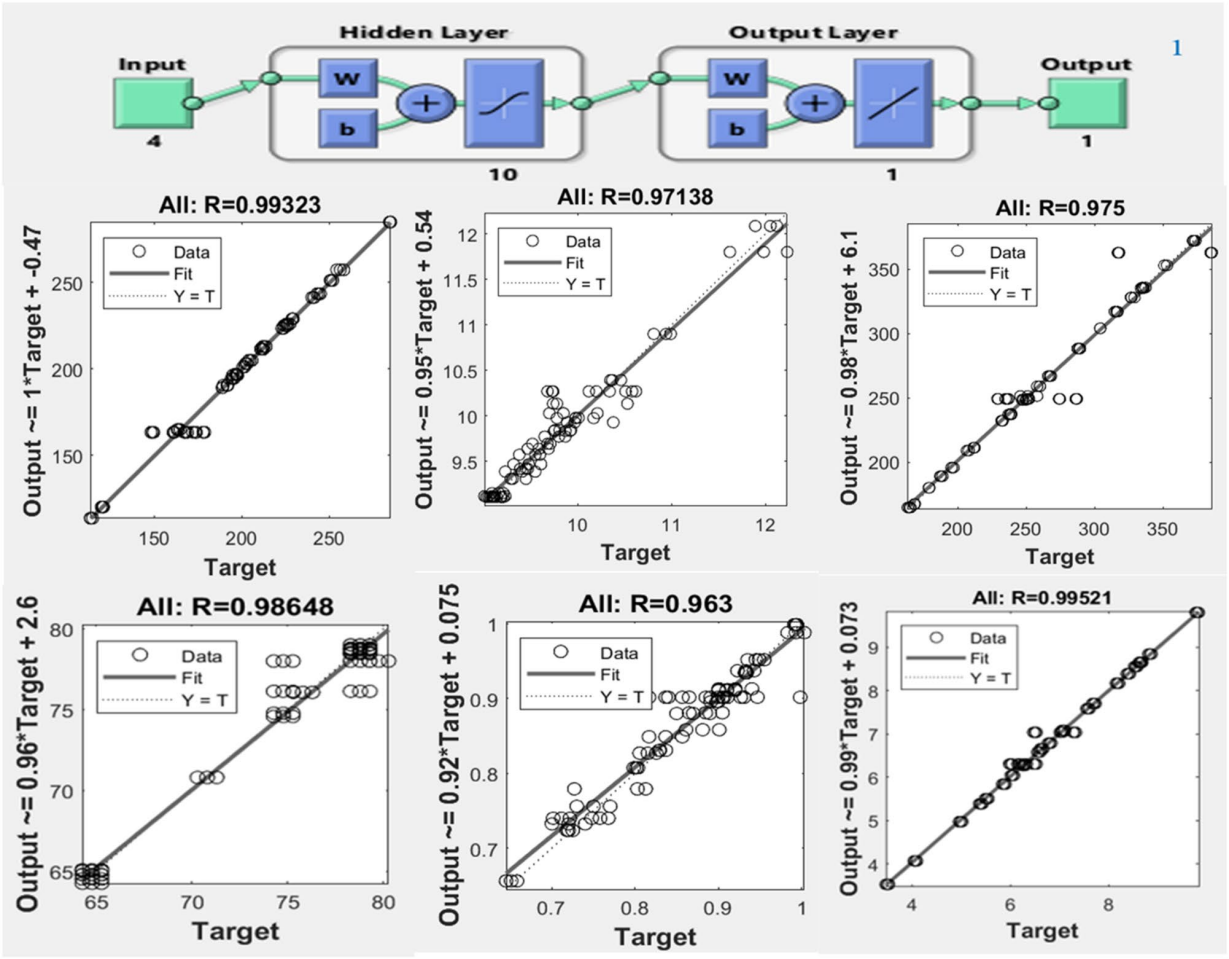
ANN architectural structure and regression plots for overall process.

The study utilized a feed-forward and BP algorithm, and several iterations of ANN prediction were carried out for all responses in order to obtain the best values. The ANN inputs were chosen based on training, validation, and testing for the impregnated state. The architectural structure of an artificial neural network for each of the six responses and the regression plots are shown in Fig. 3. The experimental values of the response variables were reasonably predicted using the ANN model. The R^2^ value showed that the predicted and necessary values were typically very similar. The higher the correlation coefficient, the better the model fits the data and the more precisely processing conditions may be predicted^29,30^.

### Comparison with RSM

The comparison has been done among the predictions of trained ANN and RSM models to the experimental results of different responses in vacuum impregnated samples. The prediction performance of RSM and ANN models was compared using statistical parameters including R^2^, SSE, chi-square, HYBRID, MPSD, RMSE, and ARE. The artificial neural network matches the data points more closely than the RSM model. The performance of ANN is backed by its higher R^2^, lower chi-square, HYBRID, MPSD, RMSE, and ARE values (Table 3).

**Table 3:** Performance of RSM and ANN models using statistical error parameters.

Statistical function						
Model	TPC	TFC	AsA	TCC	FRSA	Carotenoid
ERSS						
RSM	4.0401	0.1024	4.0000	0.0000	0.9600	0.0010
ANN	0.2907	0.0162	3.3804	0.0016	0.1130	0.0477
χ2						
RSM	0.0007	0.0004	0.0005	0.0000	0.0000	0.0000
ANN	0.0000	0.0001	0.0004	0.0000	0.0029	0.0002
HYBRID						
RSM	0.0012	0.0040	0.0009	0.0008	0.0000	0.0002
ANN	0.0003	0.0016	0.0009	0.0058	0.0042	0.0039
MPSED						
RSM	0.0065	0.0212	0.0049	0.0041	0.0002	0.0010
ANN	0.0218	0.0268	0.0130	0.0127	0.0219	0.0139
RMSE						
RSM	0.3732	0.0594	0.3714	0.0008	0.0000	0.0019
ANN	0.1001	0.0236	0.3414	0.0058	0.0042	0.0405
ARE						
RSM	0.0012	0.0039	0.0009	0.0008	0.0000	0.0002
ANN	0.0003	0.0015	0.0008	0.0056	0.0040	0.0038
R^2^						
RSM	0.95	0.93	0.92	0.92	0.94	0.950
ANN	0.99	0.97	0.98	0.96	0.99	0.99
Error validation for RSM optimization						
RMSE	1.13	0.32	0.27	0.08	0.44	0.29
χ2	0.0044	0.0093	0.0002	0.0093	0.0024	0.0097

The ANN’s superior prediction accuracy can be traced back to the fact that it can estimate the system’s nonlinearity on a vast scale, whereas the RSM is limited to a second-order polynomial ^31,32^. However, the individual and interaction effects of independent variables were obtained only by RSM. Additionally, ANN is independent of the experimental environment and can include new experimental data to improve the model^32–34^. This study shows that the ANN model, which considers the nonlinear behaviour of the data set, has a better ability to fit the data.

### Optimisation of vacuum impregnation process parameters for broccoli

By comparing predicted values for optimal conditions with experimental values acquired by experimenting at the optimized combination, the effectiveness of vacuum impregnation optimization was statistically assessed. The optimized values of the independent variables include the vacuum pressure of 0.2 bar, 11 min of vacuum time, 12 min of restoration time, and 1.5% concentration of solution were anticipated to be the ideal conditions for VI of broccoli with a desirability value of 0.89. The values of TPC, TFC, AAC, TCC, FRSA, and CC values obtained at optimized conditions of independent variables were 291.20 mg GAE/100 g, 11.29 mg QE/100 g, 350.81 mg/100 g, 1.21 mg/100 g, 79.77 mg, and 8.51 mg, respectively., The model’s performance was confirmed comparing RMSE and Chi-square (Table 3).

### Analysis of vacuum impregnated broccoli florets

The broccoli samples treated under optimized conditions of the VI experiment were examined for FTIR, SEM, and XRD in order to understand the process and its effect on the microstructure and morphology of in comparison with the untreated sample.

### Fourier transform infrared spectroscopy (FTIR)

**Figure 4:**
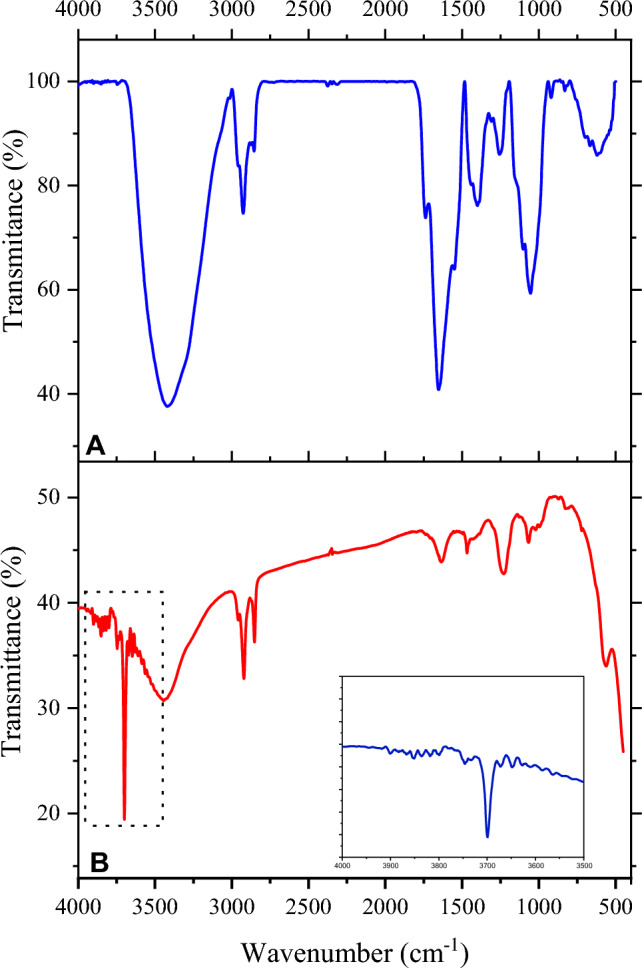
FTIR spectra of (**A**) Untreated (**B**) Treated samples.

The FTIR spectrum of the control and impregnated broccoli florets represented in Fig. 4 shows the absorption bands at different wavelengths reflecting their functional groups. Analysis performed at spectrum of mid-IR region, which is divided into four regions: (i) the single bond region 2500–4000 cm^−1^ single bond, (ii) the triple bond region (2000–2500 cm^−1^), (iii) the double bond region (1500–2000 cm^−1^), and (iv) the fingerprint region (600–1500 cm^−1^)^35,36^. In Fig. 4 (A), the FTIR spectrum for the untreated sample of broccoli shows peaks between 3600 and 3000 cm^−1^, indicating N–H stretching bands of mainly trans-ordered substructures. It shows the presence of a hydrogen bond confirming the presence of hydrate (H_2_O), hydroxyl (–OH), ammonium, or amino acids. At wavelength 3433 cm^−1^, the presence of broadband is attributed to the stretching of O–H/N–H bonds in the hydroxyl and amide groups. It confirms the presence of phenols^37^. The stretching vibrations at peak 2921 shows methylene C–H asymmetric /symmetric stretch, which indicates the presence of chlorophyll compounds in the produce. The peak at 1648 cm^−1^indicates the double bond region having alkenyl C=C stretch showing chlorophyll derivative compounds^38^. This peak shows the presence of protein (amide I) groups such as aldehydes, ketones, esters, or carboxyl components. C=O stretching vibrations at this peak caused due to aromatic ring deformations confirming the presence of flavonoids and amino acids^36^. The peak at 1397 cm^−1^ also confirms the presence of protein, as, at this level, there is CH_3_ symmetric bending (deformation) of protein. It also indicates the presence of phenol/tertiary alcohol or –OH bend in the control broccoli florets^37^. In addition, the peaks that appeared at 1262 cm^−1^ and 1260 cm^−1^ are attributed to the aromatic ethers and aryl-O stretch; it also shows the presence of organic phosphates due to P=O stretching in the region. In the bands, the peaks at 1061 cm^−1^, 914 cm^−1^, 830 cm^−1^and 620 cm^−1^ come under the fingerprint range of the band. C–O or C–C stretching and C–H bending are found in this region, indicating the presence of carbohydrates (polysaccharides cellulose), peroxides, and silicates (phosphodiester region). This region also confirms the presence of carotenoids ^39^, which is also confirmed by biochemical analysis.

The changes in the IR region (2500–4000 cm^−1^) in impregnated samples can be seen in Fig. 4 (B). Various new peaks were found in the treated samples, which may be due to the impregnation agents affecting the functional groups of the sample. The intensity of spectrum is increased at 3899 cm^−1^, 3851 cm^−1^, 3825 cm^−1^, 3818 cm^−1^, 3798 cm^−1^,3747 cm^−1^, 3699 cm^−1^, 3672 cm^−1^, 3650 cm^−1^, 3626 cm^−1^, 3566 cm^−1^, 3546 cm^−1^, 3437 cm^−1^, 3276 cm^−1^ bands compared to untreated samples. As this region is attributed to the stretching of O–H/N–H bonds, this confirm that the treated samples show an increase in phenols and flavonoids in the sample. The same is also found to be true through the biochemical analysis. The spectrum’s intensity also increased at 2924 cm^−1^ and 2854 cm^−1^, indicating the enrichment of chlorophyll content^40^. The peak at 1616 cm^−1^is related to the C=C in chlorophyll and its derivatives^38^. Peaks 1467 cm^−1^, 1224 cm^−1^,1074 cm^−1^,818 cm^−1^, and 551 cm^−1^ were seen in the treated samples but were smaller as compared to the untreated samples. The band 1467 cm^−1^ is due to bending vibration C–H and aromatic compounds related to flavonoids and aromatic rings. As per literature, hydrogen bonds found in the range 3650 and 3250 cm^−1^ should be seen in ranges 1600–1300, 1200–1000, and 800–600 cm^−1^ of IR-spectrum, indicating the presence of oxygen-containing groups, such as alcohol and phenols, which was seen in the spectrum of treated samples of our study and confirmed by biochemical analysis.

### X-ray diffraction analysis

**Figure 5:**
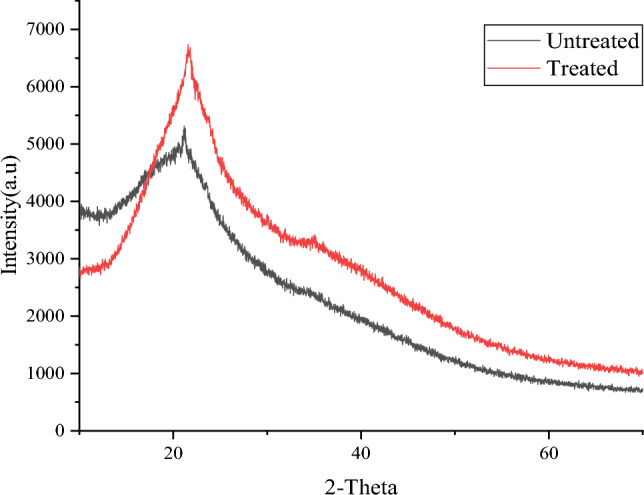
XRD graphs of Untreated and Treated sample.

X-ray diffraction analysis can determine the crystalline or amorphous nature of the powder. The results in Fig. 5 show the XRD diffractograms recorded in the scanning range of 10–75θ. The diffused curve shows that the sample is amorphous as the molecules are disorderly placed, producing dispersed bands. The diffraction patterns of both untreated and treated samples showed a broad peak around 21° and an absence of well-defined peaks. The treated samples showed more crystallinity (49.71%) than the untreated samples (38.82%), with a crystallite size of 1.123 nm for treated and 0.492 nm for untreated samples. The dispersed bands produced in the graph suggested that the sample’s molecules lacked orientation and were placed disorderly, suggesting the samples were in an amorphous state. Since no prominent peaks were observed, it can be interpreted that the chemical compounds of the sample were preserved in the treated samples and were not crystallized. The shift in the peak intensity of the treated sample suggests an increase in the compound of the treated samples, which is also confirmed in the results obtained from the FTIR and biochemical analysis^16^.

### Scanning electron microscopy

**Figure 6:**
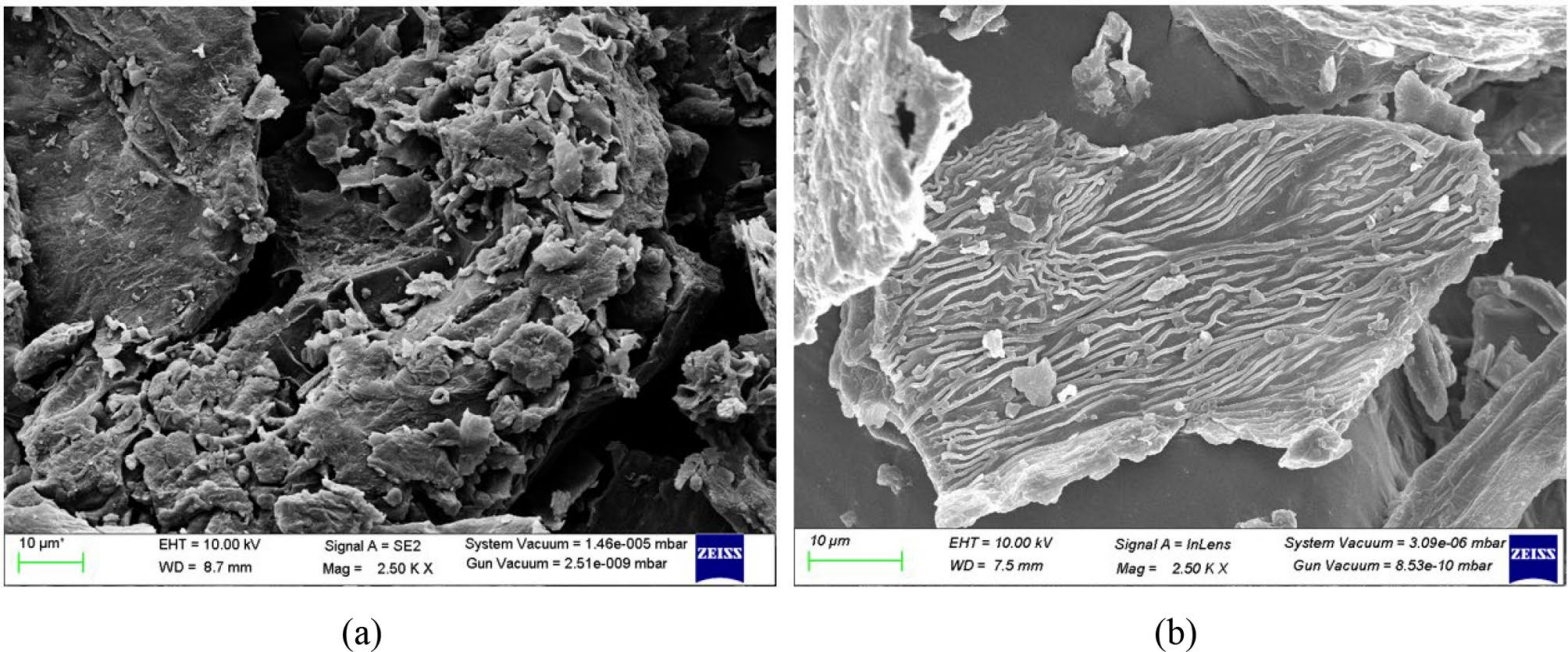
SEM Images of (**a**) Untreated and (**b**) Treated sample.

Macroscopic images of freeze-dried untreated and impregnated broccoli florets are shown in Fig. 6. It is visible that the untreated sample didn’t have a specific defined shape. In contrast, the VI treated sample showed larger cells with a more irregular shape where the VI solution penetrated into the cell provokes its swelling and the crushing of the plasmatic membrane towards the cell wall. Along with that smooth surface and lesser pore spaces were seen in the impregnated sample. It can be said that the space between the plasma lemma and the cell wall may have been filled completely with the solution through the permeable cell wall to replace intracellular air and water vacuoles leading to new structural formation. Solid gain is primarily determined by the microstructure of food tissue, the vacuum resulted in a more substantial structural change^41^.

## Discussion

Vacuum impregnated broccoli florets resulted in noticeably higher contents of biochemical compounds. When vacuum is applied to the plant tissue it causes stress within as abiotic stress. Abiotic vacuum stress may have caused changes in the microstructure, making room for increased content. Plant tissue reacts to the stress by activating biosynthetic pathways of bioactive secondary metabolites as defense response. These stress triggers the shikimic acid-phenylpropanoid metabolism which is a transduction pathway contributing to the accumulation and biosynthesis of phenolics and flavonoids^21^. The key regulatory enzymes involved in the metabolism are phenylalanine ammonia-lyase (PAL) and tyrosine ammonia-lyase (TAL). These enzymes might have been stimulated by impregnation treatment, increasing TPC and TFC. Similar outcomes for bitter gourd, capsicum, and Cornelian cherries have also been reported^27,42,43^. The reduction of enzymatically formed *o*-quinones to their precursor phenolic substrates by ascorbic acid impregnation may also be the reason of increasing phenolic content of the produce^44^. As a result, it may be argued that the impregnation agent’s impact on the biochemical content was synergistic. A higher TPC and TFC content reflected in the antioxidant activity may follow an increase in PAL activity due to an increase in FRSA^45^. It was observed that the majority of the antioxidant activity of plants is derived from phenols, which may be correlated with antioxidant capacity^46^. Phenols are composed of an aromatic ring with one or more hydroxyl substituents. This sort of molecule possesses antioxidant activity due to its ability to scavenge free radicals, donate hydrogen atoms or electrons, or bind metal cations. Another reason for increase in antioxidant property of broccoli after impregnation can also be attributed to the elevated levels of chlorophyll, which acts as an antioxidant^47^. Chlorophylls and their derivatives have shown important health-promoting functions, showing anti-mutagenic, anti-cancer, and anti-inflammatory activities. Ascorbic acid prevents chlorophyll degradation and neutralizes superoxide radicals, thereby indirectly increasing the chlorophyll content. Singlet oxygen is produced when chlorophyll excites energy directly into singlet oxygen during the Mehler reaction or by exciting photosystem I into singlet oxygen through chlorophyll activity^48^.

## Conclusions

Vacuum impregnation (VI) enhances food products’ quality with beneficial nutrients and changes their sensory and physicochemical attributes. In present work, ascorbic acid and calcium chloride solutions were used for impregnating broccoli florets. The results of the biochemical analysis showed enhancement in the total phenolic content, total flavonoid content, ascorbic acid content, total chlorophyll content and free radical scavenging activity. The same was also supported by the results of FTIR, and XRD analysis. The developed ANN model, which considers the nonlinear behavior of the data set, was found suitable in predicting the biochemical parameters of vacuum impregnated broccoli samples. Goal based optimization of the vacuum impregnation process parameters was done keeping in view of the values of quality attributes. The optimized solution of independent variables was validated by running the experiment at that condition and calculating the error terms. The SEM Images also supported the changes that occurred during the impregnation process. Thus, vacuum impregnation with calcium chloride and ascorbic acid is an effective technique to enhance the quality attributes of the broccoli florets. Further, we recommend that future research endeavors should consider use of other potential agents for vacuum impregnation of broccoli in order to enhance its nutritional parameters and well as shelf-life. It is also suggested to integrate HPLC analysis to complement the current findings and offer a more accurate and comprehensive understanding of the impact of VI technology on the phytochemical properties of broccoli.

## Data Availability

The datasets generated during and/or analysed during the current study are available from the corresponding author on reasonable request.
